# Dimensional structure of the oral health-related quality of life in healthy Spanish workers

**DOI:** 10.1186/1477-7525-8-24

**Published:** 2010-02-21

**Authors:** Javier Montero, Manuel Bravo, María-Purificación Vicente, María-Purificación Galindo, Joaquín F López, Alberto Albaladejo

**Affiliations:** 1 Department of Surgery. University of Salamanca. Salamanca. Spain; 2 Department of Preventive and Community Dentistry. University of Granada. Granada. Spain; 3 Department of Biostatistics. University of Salamanca. Salamanca. Spain

## Abstract

**Background:**

Oral health-related quality of life (OHQoL) is conceived as a multidimensional construct. Here our aim was to investigate the dimensional structure of OHQoL as measured by the Spanish versions of the Oral Impacts on Daily Performance (OIDP) and the Oral Health Impact Profile (OHIP-14) questionnaires applied simultaneously.

**Methods:**

We recruited a consecutive sample of 270 healthy Spanish workers visiting the Employment Risk Prevention Centre for a routine medical check-up. OHIP-14 was self-completed by participants but the OIDP was completed in face-to-face interviews. An Exploratory Factor Analysis (EFA) was performed to identify the underlying dimensions of the OHQoL construct assessed by both instruments. This factorial structure was later confirmed by Confirmatory Factor Analysis (CFA) using several estimators of goodness of fit indices.

**Results:**

EFA and the CFA identified and respectively confirmed a set of 3 underlying factors in both questionnaires that could be interpreted as functional limitation, pain-discomfort, and psychosocial impacts. The model achieved was seen to fit properly for both instruments, but the factorial structure was clearer for the OIDP.

**Conclusions:**

The results provide evidence for construct equivalence in the latent factors assessed by both OIDP and OHIP-14, suggesting that OHQoL is a three-dimensional construct. The prevalence of impact on these three factors was coherent between both indicators, pain-discomfort having the highest prevalence, followed by psycho-social impact, and functional limitation.

## Background

Oral health-related quality of life (OHQoL) is a multidimensional construct that refers to the extent to which oral problems disrupt an individual's normal functioning [1,2]. The multidimensional nature of OHQoL is also recognized in the most widely accepted theoretical model of oral health reported by Locker [3], which postulates that there are five consequences of oral disease (impairment, functional limitation, pain/discomfort, disability, and handicap) and that these are related sequentially. Consequently, all OHQoL indicators group their items within different topic categories, but the number and nature of these categories vary across instruments. Moreover, the assignment of items within the dimensions of OHQoL indicators are mostly based on authors' expert knowledge of the theoretical framework. However, some statistical methods, such as exploratory and confirmatory factor analyses, are mandatory for exploring the underlying multivariable relationships and could be helpful in building up a picture of what is really being measured.

Using principal component factor analysis, some authors have considered OHQoL in adults or the elderly as a single construct [4,5]. In contrast, however, others have identified a range of three-to-five latent dimensions related to physical, psychological and social performance in the OHQoL construct of adults or the elderly [6-8].

One recent European project [9] has recommended focusing on three major OHRQoL indicators: OHIP-14 [10], OHQoL-UK [11] and OIDP [12]. Of these, the two most widely used and internationally accepted are OHIP-14 and OIDP. Both instruments are based on Locker's well-established conceptual model [3] and have recently been validated in Spain [13,14].

Whereas the psychometric properties of both instruments (reliability and validity) have been found to be satisfactory in a variety of cultural contexts, the dimensional structure of both indicators is still a controversial issue, and presumably both of them should measure the same construct from different perspectives: one using a severity-based approach (OIDP) and the other using a frequency-based approach (OHIP) for summarizing the perceived impacts on the OHQoL. It would also be desirable to identify a set of core constructs for cross-cultural comparisons of oral wellbeing or to shorten the questionnaires available on the basis the major dimensions detected.

Based on our previous experiences [13,14], we hypothesized that oral health-related quality of life, in spite of being a single construct, could comprise at least 3 dimensions conceived as pain-discomfort, eating performance and aesthetics because we had observed that individuals seemed to understand these dimensions to be distinct aspects of oral wellbeing. For example, visibly stained teeth could only affect the aesthetic dimension but not the other two; shortened dental arches could only affect eating performance but not the other two, and sensitive teeth could only affect the pain-discomfort dimension but not the other two. Of course, several clinical conditions could partially or totally impinge on these dimensions.

The present work aims to identify the dimensional structure of OHQoL in a healthy Spanish workers by applying Confirmatory Factor Analysis to these two widely accepted instruments.

## Methods

### Study design

A cross-sectional epidemiological study was performed in the City of Granada and its province. A consecutive sample of 295 healthy workers visiting the Employment Risk Prevention Centre for a routine medical check-up were invited to take part in the study, 270 of whom finally participated in the study (91.5%), although the drop-outs were similar in terms of their socio-demographic characteristics. All interviewees were briefed about the purpose of the study and written consent was sought for questionnaire-led interviews and simple oral examinations. Individuals younger than 25 years of age or seeking dental treatment were excluded, because we wished to assess the construct of OHQoL in a mature dental population with no acute oral problems in order to obtain a baseline picture of the construct in this sample, which could be compared in the future with some other sociodemographic profiles of adults or even with a representative sample of the Spanish population.

### Instruments

The OHIP-14 (Oral Health Impact Profile) comprises 14 items that explore seven dimensions of impact (functional limitation, pain, psychological discomfort, physical disability, psychological disability, social disability, and handicap) and participants respond to each item according to the frequency of impact on a 5-point Likert scale ranging from *never *to *very often *(never = 0, hardly ever = 1, occasionally = 2, fairly often = 3, very often = 4), using a twelve-months recall period.

In the original development of this instrument, factor analysis revealed a single underlying factor that accounted for almost the 70% of the variance [10]. However, later research performed in Germany using the extended version reported a parsimonious set of dimensions termed oral functions, pain, and psychosocial impact [8].

The OIDP (Oral Impacts on Daily Performances) questionnaire assesses the impacts of oral conditions on the abilities of individuals to perform eight daily activities. For each dimension (eating, speaking, hygiene, occupational activities, social relations, sleeping-relaxing, smiling, and emotional state), the severity and either the frequency or duration of each impact are recorded on a Likert scale. Firstly, individuals responded whether or not problems with the mouth, teeth or dentures had caused them any difficulty with each of the eight activities in the past six months. If the answer was "no" the item score was coded as "0", and we enquired as to the presence of difficulty with the next item. However, if the answer was "yes", the frequency and severity of this difficulty had to be assessed. Frequency had to be recorded only if the subject had this difficulty on a regular basis over the past six months, being coded as follows: less often than once a month = 1; about 1-2 times a month = 2; about 1-2 times a week = 3; about 3-4 times a week = 4; every day or nearly every day = 5. Nevertheless, if individuals perceived that this difficulty to affected them only for a part of this 6-month period, then the duration of this event was recorded, coding the responses as follows: for 5 days or less = 1; for more than 5 days, up to a month = 2; for more than 1, up to 2 months = 3; for more than 2, up to 3 months = 4; for more than 3 months = 5. Then, individuals expressed how much effect the difficulty had on their everyday life, coding the responses as follows: no effect = 0; a very minor effect = 1; a fairly minor effect = 2; a moderate effect = 3; a fairly severe effect = 4, a very severe effect = 5.

This instrument has commonly been applied as a one-dimensional construct, in terms of a single OIDP summary score, but recently a three-dimensional structure (designated as physical, psychological and social impacts) has been confirmed statistically [15-17].

Here, the OHIP was self-completed by participants in a waiting room, whereas the OIDP was completed in face-to-face interviews in a quiet private room by a trained and calibrated examiner (MJ) to overcome the complexities of the instrument. Furthermore, these were the administration methods recommended by the original authors [10,12] and we therefore considered them to be the best approach to detect the underlying dimensions of the construct. The examiner ensured full completion of the OHIP-14, before starting the interview with the OIDP.

In both instruments, an additive total scoring method was used. For the OHIP, it was calculated by summing the item codes for the 14 items. For the OIDP, total impact was quantified by summing the item scores, which were obtained by multiplying the frequency and severity scores for each of the eight items, and converting this total score into a percentage format. This scoring system yields an intuitive oral impact score. The frequency and severity scores are Likert-type scales, but a zero score is only possible for severity. Hence, severity is weighted and can produce a zero score for an item-related impact if the individual considers that there is no effect on daily life activities.

To estimate the prevalence of impacts, the presence of any impact was recorded for each measure or domain. For OHIP, an impact was recorded as present if it was reported at the threshold of "occasional" or more frequently (≥2 on the 5-point Likert scale). For OIDP, an impact was considered if it was recorded at a moderate or more severe level (≥3 in the 6-point Likert scale).

### Data analysis

An Exploratory Factor Analysis (EFA) was performed on one half of the sample (n = 135) to identify the latent dimensions of OHQoL. Factors with an eigenvalue of less than 1 were disregarded. A varimax rotation was conducted to achieve a simpler structure. Items were assigned to the rotated factors when they had a loading of 0.5 or higher on a single factor [18].

Later, Confirmatory Factor Analysis (CFA) was applied to the data from the other half of the sample to verify the factor structure. The goodness-of-fit of the model to the data was evaluated using the following parameters. The Chi-square test, which indicates the amount of difference between expected and observed covariance matrices. A Chi-square value close to zero indicates little difference between the expected and observed covariance matrices. In addition, the probability level must be greater than 0.05 when Chi-square is close to zero. Equivalently, Chi-Square/DF ≥ 3 indicates an unacceptable model fit, although this index is strongly influenced by sample size [19]. The comparative fit index (CFI) is equal to the discrepancy function adjusted for sample size. The CFI ranges from 0 to 1, a higher value indicating better model fit. An acceptable model fit is indicated by a CFI value of 0.90 or greater [20].

The Root Mean Square Error of Approximation (RMSEA) is related to the residual error in the model. RMSEA values range from 0 to 1, a smaller RMSEA value indicating a better model fit. An acceptable model fit is indicated by an RMSEA value of 0.06 or less [20]. To evaluate the statistical signification of RMSEA, the "p-close" value has been proposed [21]; that is, the p-value to test the null hypothesis (RMSEA ≤ .05). An acceptable value of p-close should be >0.05.

An overall conclusion about the fit of each model can be obtained by considering these indices simultaneously, as recommended by Schermelleh-Engel *et al*. [22], and by obtaining at least three fit statistics indicating an acceptable fit.

Once the factorial structure has been confirmed, the parameter estimates are examined as follows: the critical ratio (CR) of each parameter estimate divided by its standard error is distributed as a z statistic and is significant at the 0.05 level if its value exceeds 1.96, and at the 0.01 level if its value exceeds 2.56 [23].

EFA was performed with the Statistical Package for the Social Sciences (SPSS v.15) whereas CFA was performed with the AMOS computer software program, version 7.0 [24].

## Results

### Sample profile

Since the factorial structure might vary across the gender and socio-demographic characteristics of a population, and since this is of importance when it comes to designing intervention programs, it is necessary to describe the study sample (Table 1). The mean age of the participants was 45.2 ± 9.5 yrs (χ ± SD): 45.6% were male; 83.3% were non-manual workers, and 57% lived in the City of Granada. In behavioural terms, 93% of subjects brushed their teeth at least once a day and 36.3% routinely visited their dentist at least once a year. On clinical examination, most participants had a good state of oral health. The sample had a mean of 26.4 ± 4.2 standing natural teeth, with 17.8 ± 5.6 healthy non-restored teeth. The decayed, missing and filled teeth index (DMFT) was 10.7 ± 5.0, of which a mean of 3.2 ± 2.5 teeth were decayed; 3.3 ± 3.7 were missing, and 4.3 ± 3.5 were filled. The periodontal status afforded a CPI score of zero in 3.1 ± 2.2 of sextants. More than 90% of the subjects were dentate without dentures.

**Table 1:** Sociodemographic, behavioural and clinical description of the sample (n = 270).

SOCIODEMOGRAPHICS VARIABLES	n	%
Sex		
		
Male	123	45.6
Female	147	54.4
		
Social Class^a^		
		
High	113	41.8
Medium	112	41.5
Low	45	16.7
		
Residence		
		
Urban	154	57.0
Rural	116	43.0

Age (Mean ± SD)	45.2 ± 9.5	

<34 yrs	39	14.4
35-44 yrs	85	31.5
45-54 yrs	99	36.7
55- 65 yrs	47	20.4

BEHAVIOURAL VARIABLES	n	%

Brushing habits		
		
2-3 times/day	181	67.0
Once/day	70	25.9
Less than once/day	19	7.0
		
Dental visit pattern		
		
Check-up visits	98	36.3
Problem-based visits	172	63.7
		
CLINICAL VARIABLES	Mean ± SD	

Prosthodontic variables		

Missing teeth	3.3 ± 3.7	
Replaced teeth	1.3 ± 2.8	
Occlusal Units	6.4 ± 2.2	
Aesthetic Units	5.7 ± 1.0	
Standing teeth	26.4 ± 4.2	
Number of replaceable teeth	1.4 ± 2-2	

Caries variables		

Decayed teeth	3.2 ± 2.5	
Healthy restored teeth	4.3 ± 3.5	
DMFT (Decayed Missing and Filled Teeth)	10.7 ± 5.0	
Healthy non-restored teeth	17.8 ± 5.6	

Periodontal variables^b^		

Sextants with CPI = 0	3.1 ± 2.2	
Sextants with CPI = 1	0.9 ± 1.4	
Sextants with CPI = 2	0.5 ± 0.8	
Sextants with CPI = 3	1.1 ± 1.6	
Sextants with CPI = 4	0.1 ± 0.5	

^a ^Social Class was estimated in occupational terms as follows: High: skilled non-manual worker; Medium: skilled manual worker; Low: non-skilled manual worker.

^b ^CPI: Community Periodontal Index

SD: Standard deviation

### Factorial Structure

For both indicators, the measurement of sampling adequacy (Kaiser-Meyer-Olkin) and significance level of Bartlett's test of sphericity (p-value < 0.001) indicated that there were probably significant relationships among items, and that the data were suitable for factor analysis (Table 2 and 3). For both indicators, three components with eigenvalues above 1 emerged from the factorial analysis and were supported by the elbow in the corresponding scree plot of eigenvalues. In the OIDP, these three factors explained 64.3% of the total variance (Table 2), while in the OHIP-14 they explained 58.1% (Table 3). The factor loadings are depicted in the rotated component matrix. For the OIDP, the first factor (termed "Functional Limitation") comprised items related to *speaking, hygiene, occupational *and, partially, to *eating*. Factor 2 (designated "Psychosocial Impact") comprised *social relations *and *smiling*. Factor 3 (labelled as "Pain-discomfort") comprised the items referred to *sleeping-relaxing*, *emotional state *and, partially to *eating*. In the OHIP-14, the same factors emerged from the rotated matrix: Factor 1 represented the "Psychosocial impact"; Factor 2 the "Pain-discomfort", and Factor 3 the "Functional limitation". Item 5 (*Self-consciousness*) had a mixed load between Psychosocial and Pain-discomfort factors. All factors in both indicators had alpha values ranging between 0.46 and 0.84.

**Table 2:** OIDP Item Loadings > 0.50 from Exploratory Factor Analysis followed by Varimax Rotation

ITEMS	Factor 1	Factor 2	Factor 3
Speaking	0.75		

Cleaning	0.65		

Occupational	0.80		

Social relations		0.83	

Smiling		0.83	

Eating	0.40		0.56

Sleeping-relaxing			0.83

Emotional state			0.69

Eigenvalues	2.9	1.3	1.1

Variance explained	22.9	20.9	20.4

Cumulative variance	22.9	43.9	64.3

Alpha	0.60	0.71	0.60

Kaiser-Meyer-Olkin measure of sampling adequacy: 0.72

Bartlett's test of sphericity: χ^2^, d.f; p-value = 454.04, 28; p < 0.001

**Table 3:** OHIP Item Loadings > 0.50 from an Exploratory Analysis followed by Varimax Rotation

ITEMS	Factor 1	Factor 2	Factor 3
OHIP-1: Speaking			0.78

OHIP-2: Sense of taste			0.78

OHIP-3: Painful aching		0.76	

OHIP-4:Uncomfortable eating		0.86	

OHIP-5: Self-conscious	0.54	0.42	

OHIP-6: Tension	0.68	0.38	

OHIP-7: Unsatisfactory diet	0.32	0.67	

OHIP-8: Interrupt meals		0.61	

OHIP-9: Difficult to relax	0.60		

OHIP-10: Embarrassed	0.71		

OHIP-11: Irritable	0.66	0.36	

OHIP-12: Occupational	0.61		0.32

OHIP-13: Unsatisfactory life	0.79		

OHIP-14: Unable to function	0.70		

Eigenvalues	3.8	2.8	1.6

Variance explained	27.2	19.7	11.2

Cumulative variance	27.2	46.9	58.1

Alpha	0.84	0.78	0.46

Kaiser-Meyer-Olkin measure of sampling adequacy: 0.89

Bartlett's test of sphericity: χ^2^, d.f; p-value = 1484.49, 91; p < 0.001

### Fit Statistics

The CFA carried out indicated an excellent fit of the model for the OIDP [χ2/d.f= 1.40, p= 0.13, CFI= 0.99, RMSEA= 0.04, p-close= 0.66] and an acceptable fit of the model for the OHIP [χ2/d.f= 2.09, CFI= 0.95, RMSEA= 0.06, p-close= 0.06]. The null hypothesis that this model fits the data was confirmed. Considering the ratio of the Chi-square statistic to the accompanying degrees of freedom, a ratio of 1.40 for the OIDP and 2.09 for the OHIP (both < 3) were considered to represent acceptable model fits. Moreover, the root mean square error of approximation (RMSEA) for both instruments was greater than the 0.06 criterion (p-close also >0.05) and, additionally, the Comparative Fit Index (CFI) value met the criterion (0.90 or larger) for acceptable model fit.

Thus, the CFA analysis confirmed the three-factor structure for both the OIDP (Table 4) and the OHIP-14 (Table 5), and all parameter estimates for the confirmatory factor model were significant at the 0.001 level. While unstandardized parameter estimates retain scaling information of variables and can only be interpreted with reference to the scales of the variables, standardized parameter estimates are transformations of unstandardized estimates that remove scaling and can be used for informal comparisons of parameters within the model. Thus, standardized estimates correspond to effect-size estimates. Table 4 shows that regarding the factor termed "Functional Limitation", the *occupational *item is the most relevant one, followed sequentially by *speaking *and *cleaning*. Within the dimension termed "Psychosocial impact" the *social *item is the most related one, and within the dimension termed "Pain-discomfort", the *sleeping-relaxing *item is the strongest factor-related one. Likewise, Table 5 shows that regarding the latent factor termed "Psychosocial impacts", item 6 (*tension*) is the most related, followed by item 11 (*irritable*) while, by contrast, the least related is item 14 (*unable to function*). With regard to factor 2 termed "Pain-discomfort", the most related item is OHIP-8 (*interrupt meals*), followed by item 4 (*uncomfortable eating*). For the third dimension, called "Functional Limitation", the item with the greatest weight is item 1 (*speaking*).

**Table 4:** Parameter estimates of unstandardized and standardized regression weights for the three-factor model of the OIDP.

Item	FACTORS	Unstandardized Regression Weights	S.E.	C.R.	p-value	Standardized Regression Weights
Speaking	Functional limitation	1.00				0.69
Cleaning		0.80	0.146	5.53	***	0.40
Occupational		1.05	0.138	7.59	***	0.81

Social	Psychosocial Impact	1.00				0.67
Smiling		1.00				0.84

Eating	Pain-Discomfort	1.00				0.58
Emotion		0.94	0.151	6.23	***	0.58
Sleep-relax		1.03	0.169	6.12	***	0.60

S.E. Standard error. C.R. Critical ratio

All items are statistically significant. p =*** means p < 0.000

**Table 5:** Parameter estimates of unstandardized and standardized regression weights for the three-factor model of the OHIP-14.

Item	FACTORS	Unstandardized Regression Weights	S.E.	C.R.	p-value	Standardized Regression Weights
OHIP5	Psychosocial Impacts	1.00				0.65
OHIP6		1.03	0.095	10.85	***	0.81
OHIP9		0.92	0.098	9.43	***	0.67
OHIP10		0.71	0.079	9.04	***	0.64
OHIP11		0.74	0.073	10.17	***	0.74
OHIP12		0.38	0.043	8.71	***	0.61
OHIP13		0.57	0.061	9.46	***	0.68
OHIP14		0.18	0.023	7.64	***	0.53

OHIP3	Pain-discomfort	1.00				0.53
OHIP4		1.57	0.163	9.68	***	0.71
OHIP7		1.18	0.159	7.40	***	0.71
OHIP8		1.04	0.138	7.52	***	0.74

OHIP1	Functional limitation	1.00				0.66
OHIP2		1.00				0.47

S.E. Standard error. C.R. Critical ratio

All items are statistically significant. p =*** means p < 0.000

**Figure 1:**
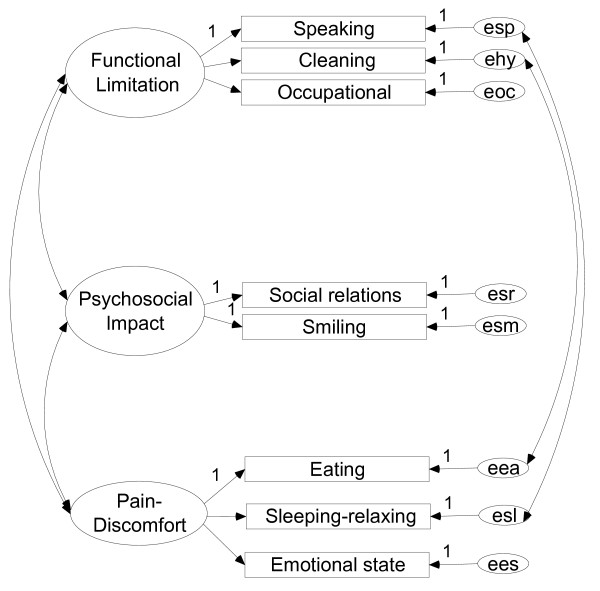
**Hypothesized three-factor model of the eight-item Oral Impacts on Daily Performances (OIDP)**. Random measurement errors denoted as esp, ...,ees, respectively

**Figure 2:**
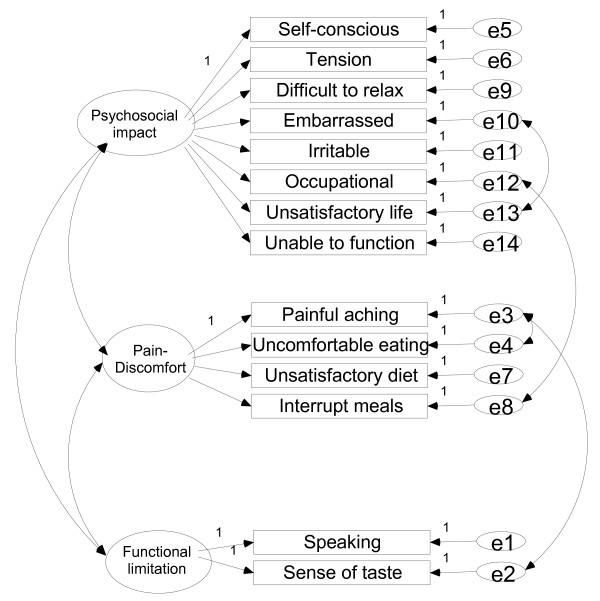
**Hypothesized three-factor mode of the 14-item Oral Health Impact Profile (OHIP-14)**. Random measurement errors denoted as e1-e14.

The proposed models for OIDP and OHIP-14 are depicted in Figures 1 and 2 respectively. In these models, a residual relationship between dimensions can be observed through some items: i.e., in the case of the OIDP some interaction is observed between eating and hygiene items and between the sleeping-relaxing and speaking items for the OIDP (Figure 1). The residual relationships between the OHIP items are depicted in Figure 2. For the OIDP, the inter-factor correlations between Pain and Psychosocial was 0.51; between Pain and Functional limitation it was 0.64, and between Psychosocial and Functional limitation it was 0.41. For the OHIP these correlations were 0.71, 0.59 and 0.60 respectively.

**Figure 3:**
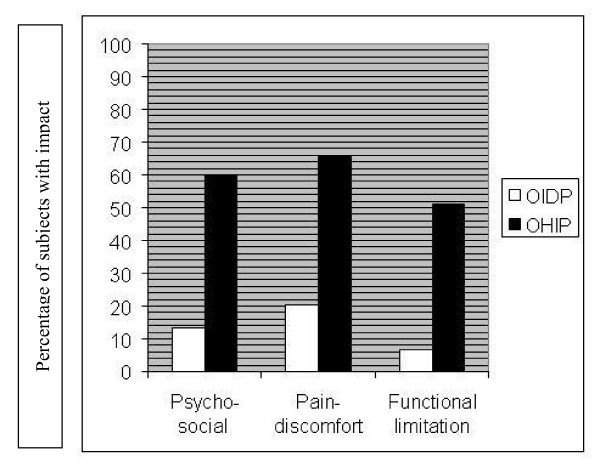
**Percentage of subjects with impact among the dimensions supported by the factorial solution using both the OIDP and the OHIP**.

Since these three factors could be conceived as matching among the indicators, an estimation of the prevalence of impact in the Psycho-social, Pain-discomfort and Functional limitations dimensions is depicted in Figure 3 using the OIDP and OHIP. A higher prevalence of impact in these dimensions can be seen when the OHIP was used than when the OIDP was employed, although there is a certain degree of harmony in the trends of prevalence of three factors in both indicators, Pain-discomfort having the highest prevalence, followed by Psycho-social impact, and Functional Limitation.

## Discussion

This study focused on exploring the dimensions of the OHQoL construct as measured by two well known instruments (OIDP and OHIP-14) in a consecutive sample of healthy Spanish workers. To our knowledge, this is the first study that has focused on exploring the factorial structure of OHQoL by using these instruments simultaneously, although some authors have analyzed dimensions using a generic instrument (such as the EQ-5D) and the OHIP-14 simultaneously in South-Australian patients [25]. They conclude that both instruments cover an overlapping domain of pain, but are discrepant as regards the specific aspects encompassed within physical, psychological and social wellbeing.

In the present study, sample size (n = 270) and the high response rate (91.5%) of this pseudo-probabilistic method of subject recruitment seem to be acceptable for such an objective. However, since perceptions of health and disability are influenced by the social, cultural and political context in which they are assessed, and since our convenience sample of healthy workers does not reflect the general Spanish population, it was considered at least necessary to check whether the dimensions identified by the EFA in half of the sample were consistently confirmed by CFA in the other half using the usual goodness-of-fit measurements. The rationale of this focuses on assuring the external validity of the dimensions initially identified.

While EFA simply requires a determination of the factor structure (model) and an explanation of the maximum amount of variance, CFA requires *a priori *specification of a model, the number of factors, knowledge of which items load on each factor, a model supported by theory and error explicitness. In our study, since no hypotheses have been consistently stated because of the lack of consensus in the literature (some researchers have identified a 3-factor structure while others have used those measures assuming only a one-factor underlying structure) the information necessary to specify the model was captured from EFA. CFA specifically, relies on several statistical tests to determine the adequacy of model fitting to the data. However, some shortcomings should be taken into account when interpreting the findings, because although this method identified the structure that best fitted the data, the fit indices did not preclude other structures from providing equally good or even better fits, and ultimately the process relied on subjective consideration of the best model, in an attempt to be coherent with the hypothesized underlying theory. In the present study, CFA was used to lend quantitative support to a qualitative interpretation.

The evidence suggests that health-related quality of life is multidimensional, including physical, psychological and social dimensions [26]. Thus, being a subset of this it should be assumed that OHQoL is also multidimensional [27]. In the present study, the CFA confirmed that a three-factor model fitted the data well, supporting the hypothesis that the construct measured by both questionnaires consists of three domains, interpreted as functional limitation, pain-discomfort and psychosocial impacts, all of them already present in the multidimensional Locker model [3] on which both instruments were based. These dimensions have been reported previously by other authors using either the extended version of the OHIP, on German adults [8], or the OIDP, on Tanzanian adults [15], or an expert-based set of items on Swedish adults [28]. Also, the same number of domains and with a similar nature have also been reported for children in Perú [16], Tanzania [17] and Hong Kong. [29].

In general there is some agreement with previous studies focusing on the dimensions measured by the OHIP-14, because pain-related items (items 3, 4, 8 and 7) and some psychosocial-related items such as items 6, 9, 12 and 13 were consistently assigned to the so-called dimensions, as reported elsewhere [25]. In contrast, the items reported here as belonging to functional limitation (item 1 and 2) were included within the Psychosocial dimension upon performing EFA [25]. Furthermore, it was found that for the OHIP-14 the first factor strongly dominated the factorial structure, although the other two dimensions were also significant.

With respect to the OIDP, a three-dimensional structure in which the *social *and *smiling *items were grouped together in the same domain was also found, as reported elsewhere [15-17]. Moreover it was also observed here that *eating*, *sleeping-relaxing *and *emotional state *shared the same factor (Table 4), as has been found for adults [15] and for children [17]. However, we have interpreted this domain as a pain-discomfort dimension while those authors interpreted it as a functional or psychological dimension respectively. Our interpretation was based on previous studies carried out on the same reference population, in which "oral pain-discomfort" was the most predominant cause of impact within those items [13]. Notwithstanding, this fact was also evident in Tanzanian children [17] in whom pain-discomfort events were the most prevalent causes of impact within all items except for *speaking*, *cleaning *and *smiling*, and mostly in the *sleeping*, *emotion*, *occupational *and *eating *items. In children [16,17]*eating *and *cleaning *were found to belong to the same physical domain. Our results also indicate that *eating *is partially loaded on the functional limitation dimension, as is *cleaning *(Table 2), and that also there is still a residual relationship between both items in the model (Figure 1), although in our setting this item loaded higher on a factor shared with *sleeping-relaxing*, as reported for adults [15].

Our findings are expected at least to contribute to an important ongoing discussion about the exploration of the dimensions of the OHQoL that will permit the development of a preliminary theory for further testing in different settings (structural reliability). It has been reported that the process of assessing the validity of OHQoL indicators should continuously evaluate the theoretical framework and the content of the construct within the natural environment of the population in question [30,31]. The theoretical background postulated that all dimensions may be disturbed sequentially; for example a pain-related condition may affect physical, psychological or social performance and may even generate handicap. Thus, the data gathered with both instruments could reflect the effect of oral conditions with multidimensional impact. In this sense, it must be accepted that OHQoL dimensions overlap to a certain degree, and hence share a considerable amount of information that could be categorized and justified for technical reasons but that ultimately reflect the notion that the main domains in OHQoL are to some extent interconnected (all inter-factor correlations reported in this study were above 0.40).

In sum, we believe that OHQoL measures refer to at least three dimensions, although since some clinical entities are able to affect several dimensions simultaneously and since all factor analysis methods are based on the intercorrelation of items, it would seem that the construct is somewhat overlapped. Nevertheless, the fact is that most oral conditions could have impact on more than one dimension. This could be why other authors have reported that only a single component emerged from their factor analyses and explained more than 60% of the variance [4,10,11], because several items may be highly correlated as a result of a common oral disease (toothache, edentulousness...). Thus, it could be recommendable to choose an oblique rotation method, as done by Bernabé *et al *[16], in which the factors are not orthogonal; that is, they are inter-correlated, which is exactly what was found in the present study and what has been discussed by several authors [15-17,25-29].

This study has some limitations, mainly with regard to the sample profile studied, because the participants (healthy Spanish workers) were not representative of the general population of similar ages. Therefore, the present findings are only valid for the group for which they were obtained and should never be extended to the adult Spanish population. Further studies are needed to corroborate our results in other, broader settings. Furthermore, studies directed toward specific oral conditions would be able to find which dimensions are mainly affected in such conditions, because it would be expected that the impact of orthodontic needs would be higher in the Psychosocial dimension than in the Pain-discomfort dimension.

## Conclusions

The present study revealed a clear distinction within the construct of the OHQoL in three qualitatively different components (Psychosocial, Pain-discomfort and Functional limitation), with high consistency, integrated within the theoretical background. Furthermore, this factorial structure seems to be shared by OIDP and OHIP. We did not undertake a factorial analysis to derive a subset of items of the OIDP and OHIP but simply to visualize and compare the underlying factors of the multifactorial construct they were measuring. Accordingly, the construct seems fairly coherent as regards both instruments and can therefore presumably be applied to other OHQoL instruments implemented among the same age-range populations.

## Competing interests

The authors declare that they have no competing interests.

## Authors' contributions

BM conceived and coordinated the study from its design to the manuscript confection. MJ carried out the study and drafted the manuscript. AA and LJ made substantial contributions to the interpretation of data. VP and GP performed the data analysis and helped to draft the manuscript. All authors read and approved the final manuscript.
